# Der Zusammenhang zwischen Wohnungsgröße und Miethöhe in Deutschland und dessen Einfluss auf die Projektentwicklung

**DOI:** 10.1365/s41056-021-00053-9

**Published:** 2021-04-19

**Authors:** Dominik Engel, Philip Gärtner, Hans-Joachim Linke

**Affiliations:** Technische Universität Darmstadt, Darmstadt, Deutschland

**Keywords:** Projektentwicklung, Wohnungsgrößenentwicklung, Mieterlös, Baukosten, Project development, Apartment size development, Rental income, Construction costs

## Abstract

Die Entwicklung der Mietpreise auf dem deutschen Wohnungsmarkt hat in den letzten Jahren zu regulatorischen Eingriffen des Gesetzgebers geführt, um die Preissteigerung zu begrenzen. Dennoch steigen die Preise in einigen Regionen weiter und stellen Marktakteure vor Herausforderungen. Insbesondere Projektentwickler im Wohnungsbau, die die zukünftige Nachfrage in ihren Entwicklungen antizipieren müssen, müssen entscheiden, welche Wohnungsgrößen zukünftig nachgefragt werden und welchen Einfluss die Entwicklung unterschiedlicher Wohnungsgrößen auf ihren Erlös hat. Diese Studie greift dazu diesen bisher in der Literatur noch allenfalls eingeschränkt behandelten Aspekt auf. Dazu wird der Trend der Wohnungsgrößenentwicklung sowohl auf Bundes- wie auch auf kommunaler Ebene anhand amtlicher Statistiken untersucht und die Erlös- und Kostenentwicklung mit Hilfe von Mietpreisstatistiken, Angebotsdaten und Experteninterviews analysiert. Der ermittelte Trend der Wohnungsgrößenentwicklung hin zu kleineren Wohnungen impliziert eine Untersuchung der sich daraus ergebenden Konsequenzen. Die Analyse der Mietpreisstatistiken und Angebotsdaten zeigt, dass dies mit höheren Erlösen pro Quadratmeter Wohnfläche einhergeht. Die Analyse der Größeneffekte auf die Kalkulation von Projektentwicklern zeigt, dass insbesondere der Flächeneffizienz und bestimmten Positionen der Baukosten frühzeitig verstärkt Beachtung zukommen sollte. Dies legt in Verbindung mit der Mietpreisanalyse die Entwicklung kleinerer Wohnungen, unter sorgfältiger Abwägung der Randbedingungen des individuellen Projektes, nahe.

## Abstract (en)

The development of rental prices on the German housing market has led to regulatory intervention by the legislator in recent years to limit price increases. Nevertheless, prices are increasing and are confronting market players with challenges. In particular, project developers in the residential construction sector, who have to anticipate future demand in their developments, have to decide which apartment sizes will be in demand in the future and what influence the development of different apartment sizes will have on their earnings. This study takes up this aspect, which has so far only been treated to a limited extent in the literature. The trend in the development of apartment sizes at both federal and municipal level will be investigated using official statistics and the development of income and costs will be analyzed with the help of rental price statistics, supply data and expert interviews. The determined trend of the apartment size development towards smaller apartments implies an investigation of the resulting consequences. The analysis of rental price statistics and rental ads shows that this is accompanied by higher revenues per square meter of living space. The analysis of size effects on the calculation of project developers shows that especially the space efficiency and certain items of the construction costs should be given increased attention at an early stage. This suggests in combination with the analysis of the rents the development of smaller apartments under careful consideration of the constraints of the individual project.

## Einleitung

Der deutsche Immobilienmarkt ist in weiten Teilen, insbesondere in Metropolregionen, seit Jahren von stark steigenden Miet- und Kaufpreisen im Wohnsegment geprägt (Statistisches Bundesamt 2019f). Diese Entwicklung stellt Eigentümer, Mieter aber auch Projektentwickler vor Herausforderungen und Chancen. Insbesondere Projektentwickler sollten aufgrund der hohen Fertigungsdauer von Immobilien Veränderungen auf dem Immobilienmarkt antizipieren können.

Bereits in dem ersten Gesetzesentwurf zur Dämpfung des Mietanstiegs auf Wohnungsmärkten aus dem Jahr 2014 weist die Bundesregierung darauf hin, dass in prosperierenden Städten, insbesondere durch die Wiedervermietung von Bestandswohnungen, die Mieten stark ansteigen und teilweise sehr deutlich über den Vergleichsmieten liegen (Bundesrat 2014). Seit vollständigem Inkrafttreten des Mietrechtsnovellierungsgesetzes im Jahr 2015 hat sich, nach einer Evaluierung des Deutschen Institutes für Wirtschaftsforschung Berlin, der Mietpreisanstieg nur moderat verlangsamt (Bundesrat 2019).

Auch das Berliner Gesetz zur Neuregelung gesetzlicher Vorschriften zur Mietenbegrenzung auf Wohnungsmärkten, besser bekannt als der „Mietendeckel“, zielt darauf ab, dem Problem steigender Mietbelastung durch eine Begrenzung der Miethöhe zu begegnen. In der Begründung des entsprechenden Gesetzesentwurfs wird insbesondere die steigende Nachfrage im Verhältnis zu einem nicht ausreichenden Angebot als Hauptursache genannt (Abgeordnetenhaus von Berlin 2019). Unter der Annahme begrenzter Wohnbauflächen liegt daher in angespannten Märkten eine Entwicklung des Marktes hin zu geringeren Wohnungsgrößen nahe, da sich so die absolute Miethöhe reduzieren ließe und Wohlfahrtsgewinne im Bereich der Wohnungswirtschaft generiert werden könnten.

Zudem begünstigt eine steigende Anzahl an Singlehaushalten sowie allgemein rückläufige Haushaltsgrößen (Statistisches Bundesamt 2019c; Breuer and Steininger 2020) die Erstellung von Wohnungen geringerer Größe, da pro Haushalt im Durchschnitt ein geringeres Budget zur Verfügung steht. Projektentwickler sollten daher die Auswirkungen, die die Entwicklung kleinerer Wohnungen verursachen, abschätzen können. Die wissenschaftliche Auseinandersetzung mit diesem Thema war in der Vergangenheit jedoch unterrepräsentiert. Schwirley (2006) weist zwar darauf hin, dass ceteris paribus kleinere Wohnungen höhere Mieten als entsprechend größere erzielen, eine Verbindung zur Projektentwicklung wird jedoch nicht gezogen. Darüber hinaus kann dieser Zusammenhang in vielen aktuellen Mietspiegeln festgestellt werden, deren Zweck jedoch nicht darin besteht, Aussagen zu Implikationen für die Projektentwicklung zu treffen.

Die aktuelle internationale wissenschaftliche Diskussion zur Projektentwicklung im Wohnungsbau befasst sich auf der anderen Seite mit Bereichen wie insbesondere der grünen Projektentwicklung, aber auch der Risikoanalyse in der Projektentwicklung. Zhang (2015) gibt beispielsweise einen Überblick über die bestehende Literatur zur grünen Projektentwicklung und stellt fest, dass diese sich vorrangig mit den Umweltaspekten befasst und andere Aspekte der Nachhaltigkeit ausgeklammert werden. Shen et al. (2017) untersuchen in diesem Feld das vorhandene Wissen bei Projektentwicklungen in Bezug auf die Einführung von umweltfreundlicher Beschaffung in China und Priess et al. (2017) stellen fest, dass die Vorteile grüner Projektentwicklung sich signifikant auf die Investitionen auswirken. Stärker wirtschaftswissenschaftlich geprägt sind die Auseinandersetzungen zur Projektentwicklung aus dem Themenbereich der Risikoanalyse. Unter anderem wenden Renigier-Biłozor und d’Amato (2017) die Realoptionstheorie auf die Bewertung von Bauerwartungsland an und Thilini und Wickramaarachchi (2019) leiten Risikofaktoren für die Projektentwicklung, allerdings gewerblicher Projekte, ab. Die bestehende Literatur liefert somit lose Anknüpfungspunkte zu den Teilbereichen dieser Studie. Arbeiten, die beide Bereich in ähnlicher Weise vereinigen und auf denen aufgebaut werden könnte, sind den Autoren jedoch nicht bekannt.

In dieser Untersuchung wird zunächst der deutsche Wohnungsmarkt in Bezug auf die Wohnungsgrößenentwicklung analysiert. Darauf aufbauend wird die Hypothese überprüft, ob, insbesondere in Regionen mit einer hohen Wohnflächennachfrage, kleinere Wohnungen mit höheren Quadratmetermieten einhergehen. Als Untersuchungsraum wurden dafür ausgewählte Städte aus der Rhein-Main-Region, aufgrund der guten Datenverfügbarkeit und der Eigenschaft als wachsendem Agglomerationsraum, herangezogen. Die für das Ergebnis des Projektentwicklers potenziell gegenläufige Entwicklung der Investitionskosten wird auf deren Abhängigkeit von der Größe der entwickelten Wohnungen hin untersucht. Hierfür werden eine ausführliche Analyse der Investitionskosten anhand der Kostengruppen der DIN 276 durchgeführt sowie Expertenmeinungen aus der Baupraxis und Projektentwicklung einbezogen. Abschließend werden die Ergebnisse zusammengefasst und kritisch gewürdigt.

## Größenveränderung auf dem deutschen Wohnungsmarkt

Im folgenden Kapitel wird überprüft, ob in Deutschland ein Trend der Wohnungsgrößenentwicklung festgestellt werden kann, da ein solcher erste Hinweise für die zukünftige Entwicklung geben könnte. Hierfür werden Zeitreihen der durchschnittlichen Wohnfläche je Baugenehmigung und im Bestand für den gesamtdeutschen Markt und den Untersuchungsraum mittels deskriptiver Statistik dargestellt. Die Darstellungen werden qualitativ erörtert.

**Figure 1:**
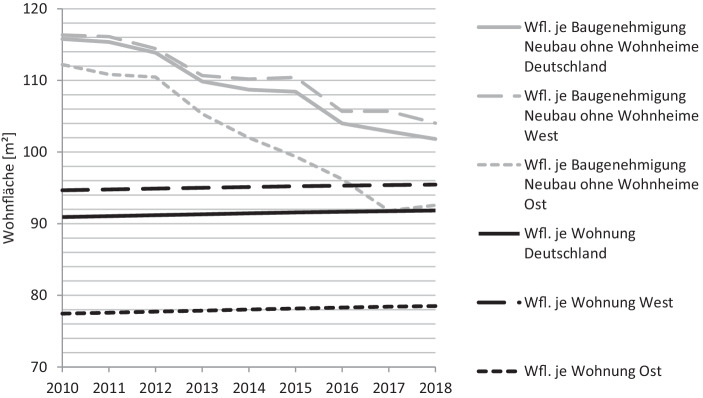

Wohnungsgrößentrends zeigen sich im Bestand aufgrund der Langlebigkeit (Smith et al. 1988) nur gedämpft und durch die hohe Produktionsdauer von Immobilien erst mit erheblicher Verzögerung (Janssen et al. 1994). Neben der Analyse des Wohnungsgrößenbestands gibt daher eine Untersuchung der Baugenehmigungen weiteren Aufschluss über aktuelle Entwicklungen. Abb. 1 zeigt sowohl die Entwicklung des Wohnflächenbestands pro Wohnung als auch die der Wohnfläche neu genehmigter Wohnungen in Deutschland.

Es wird deutlich, dass die Wohnfläche je Baugenehmigung in den letzten Jahren stark rückläufig war, was die These eines Trends hin zu kleineren Wohnungen bei Neuentwicklungen unterstützt. Es zeigt sich aber auch, dass seit 2010 ein Anstieg der mittleren Wohnfläche stattgefunden hat, da Neubauwohnungen, trotz des beschriebenen Trends, größer sind als durchschnittliche Bestandswohnungen. Das frühere Bundesgebiet und die neuen Bundesländer, einschließlich Berlin, unterscheiden sich in der Höhe der absoluten Zahlen sowohl im Bestand wie auch bei den Baugenehmigungen. Kein Unterschied ist jedoch in der Richtung des Trends in beiden Gebieten zu erkennen. Sowohl in den neuen Bundesländern als auch im alten Bundesgebiet ist eine sinkende Wohnfläche der Baugenehmigungen je Wohnungen zu beobachten. Dabei ist zu berücksichtigen, dass hier nicht zwischen Wohnungen in Ein- und Zweifamilienhäusern sowie Wohnungen in ländlichen oder städtischen Gebieten unterschieden wird. Eine Entwicklung hin zu kleineren Wohnungsgrößen könnte daher auch aus dem steigenden Anteil an Wohnungen in Mehrfamilienhäusern oder einer erhöhten Anzahl an Wohnungen in städtischen Gebieten resultieren, da diese typischerweise kleiner als ihre Pendants sind.

Für die Einschätzung des gesamten Wohnungsangebots sind die Bestandszahlen ein sinnvoller Indikator. Zur Beurteilung der Veränderung der Herausforderungen von Projektentwicklern eignen sich die Genehmigungszahlen aufgrund der Sensitivität in Bezug auf aktuelle Veränderungen jedoch besser. Insgesamt kann festgestellt werden, dass trotz unterschiedlicher Höhen der absoluten Zahlen die Entwicklung im neuen und alten Bundesgebiet in die gleiche Richtung zeigt. Sollte sich dieser Trend fortsetzen, sollten Projektentwickler zukünftig verstärkt die Entwicklung kleinerer Wohnungen berücksichtigen.

Da Projektentwicklungen insbesondere auf Wohnungsmärkten mit hoher Nachfrage nach Wohnraum durchgeführt werden, wird im Folgenden ergänzend die Wohnflächenentwicklung in exemplarischen Gebieten mit hoher Nachfrage untersucht. Nach einer Studie der Hans-Boeckler-Stiftung wies eine Wohnung in deutschen Großstädten im Jahr 2014 eine Wohnfläche von ca. 73 m^2^ auf (Lebuhn et al. 2017). Wohnungen in Großstädten sind somit unabhängig der in Abb. 1 beschriebenen Entwicklung im Durchschnitt kleiner, was aufgrund der hohen Nachfrage in diesen zumeist angespannten Wohnungsmärkten nachvollziehbar ist.

**Figure 2:**
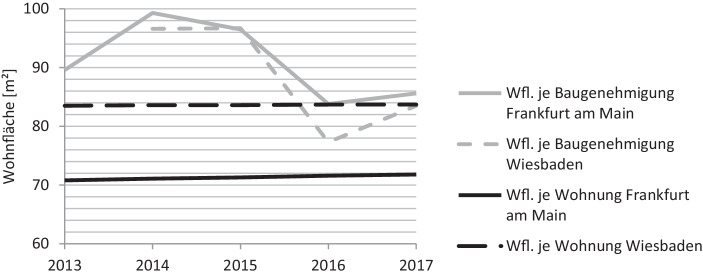

Aus dem Untersuchungsraum der Rhein-Main-Region wurden verfügbare Wohnungsbestands- und Baugenehmigungsdaten aus zwei Städten mit mehr als 100.000 Einwohnern herangezogen. Es zeigt sich in Abb. 2, dass die Wohnfläche in Frankfurt am Main und Wiesbaden je Wohnung im Zeitverlauf leicht ansteigt. Die durchschnittlichen Wohnungsgrößen der genehmigten Wohnungen unterliegen größeren Schwankungen als auf dem gesamtdeutschen Markt. Dieser Effekt wird durch den stärkeren Einfluss von Einzelprojekten auf eine kleinere Grundgesamtheit begünstigt. In den Jahren 2016 und 2017 wiesen die genehmigten Wohnungen die mit Abstand geringsten durchschnittlichen Wohnungsgrößen im Vergleich zu den übrigen erfassten Jahren auf. Dieser starke Einbruch in beiden Städten könnte aus der Unterbringung einer höheren Anzahl an Geflüchteten um das Jahr 2016 resultieren und somit nicht repräsentativ für eine weitere Entwicklung stehen (Bundesamt für Migration und Flüchtlinge 2019).

Es können somit zusammenfassend sowohl bei der Betrachtung Gesamtdeutschlands als auch bei der Betrachtung der Städte Frankfurt und Wiesbaden sinkende Wohnungsgrößen bei den Baugenehmigungen festgestellt werden. Allerdings ist nicht eindeutig, inwieweit diese eine Reaktion des Marktes auf den steigenden Nachfragedruck darstellt, da auch die Urbanisierung, der Trend zu kleineren Haushaltsgrößen und, speziell in den letzten Jahren, die Errichtung unterdurchschnittlich großer Wohnungen für Geflüchtete einen Teil zur oben beschriebenen Entwicklung beitragen. Darüber hinaus könnten private Kapitalanleger, die typischerweise andere Nachfragepräferenzen als Selbstnutzer aufweisen, insbesondere in Metropolregionen einen verzerrenden Einfluss auf die Wohnungsgrößenentwicklung ausüben.

## Entwicklung der Erlöse durch die Entwicklung kleinerer Wohnungen

Aufbauend auf der Analyse der Größentrends wird der Einfluss der Wohnungsgröße auf die Entwicklung der Erlöse von Projektentwicklern untersucht. Hierfür werden mittels deskriptiver Statistik die Entwicklungen der Mieten über die Zeit und deren Abhängigkeiten von der Größe mittels Zeitreihengraphen und Box-Plots beschrieben. In Abschn. 3.1 werden die durch die Mietspiegelerstellung bereits geglätteten Bestands- und Neuvertragsmieten genutzt, um den allgemeinen Zusammenhang zwischen Wohnungsgröße und Quadratmetermiete zu veranschaulichen. Darüber hinaus werden in Abschn. 3.2 Angebotsmieten analysiert, da diese ein aktuelleres Bild zeichnen und stärker nach Merkmalen wie Neuprojektentwicklungen differenziert werden kann. Trotz der Vorteile der Verwendung von Angebotsmieten dürfen die Unsicherheiten, die mit Angebotsdaten einhergehen und die insbesondere für Angebotskaufpreise bereits vielfach diskutiert wurden, bei der Bewertung der Ergebnisse nicht außer Acht gelassen werden (Bauer et al. 2017; Kholodilin et al. 2017; Micheli et al. 2019).

Der erzielbare Verkaufserlös und die Miethöhe sind auf dem Wohnungsmarkt eng miteinander verknüpft. Dieser Zusammenhang findet sich beispielsweise in der Investitionsermittlung von Projektentwicklern und Investoren aber auch in Wertgutachten nach Immobilienwertermittlungsverordnung im Vervielfältiger des Ertragswertverfahrens (Bundesministerium für Umwelt, Naturschutz, Bau und Reaktorsicherheit 2015; Schulte et al. 2002) und ermöglicht die Analyse der besser und transparenter verfügbaren Mieten und Mietpreisangebote ergänzend zu Kaufpreisen und Kaufpreisangeboten.

**Figure 3:**
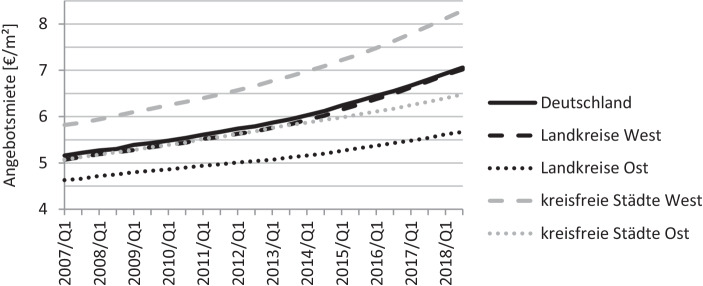

Die Richtung der Angebotsmietenentwicklung der letzten Jahre, dargestellt in Abb. 3, kann daher als Näherung für eine allgemeine Erlösentwicklung sowohl von Miet- wie auch Verkaufserlösen interpretiert werden. Die dargestellte Entwicklung bezieht sich auf hedonische Angebotsmieten für eine Referenzwohnung mittlerer Ausstattungsqualität in mittlerer Wohnlage mit 3 Zimmern, 80 m^2^ Größe und einem Alter von 30 Jahren.

Auffällig ist der deutliche Anstieg der Angebotsmieten in Deutschland innerhalb der letzten Jahre über alle dargestellten regionalen Cluster hinweg. Es zeigt sich, dass der Preisanstieg bei den kreisfreien Städten West, absolut und relativ betrachtet, mit rund 42 %, am stärksten ausfällt. Abgedämpft, aber nicht ausgeglichen, wurde der Mietanstieg durch den Anstieg des Verbraucherpreisindexes um rund 16 % von 2007 bis 2018 (Statistisches Bundesamt 2019g). Außerdem stieg im gleichen Zeitraum das durchschnittliche preisbereinigte verfügbare Einkommen um rund 10 % (Statistisches Bundesamt 2019e). Vom insgesamt verfügbaren Budget muss deutschlandweit demnach bei gleichbleibender Wohnflächeninanspruchnahme pro Person ein höherer Anteil für das Gut Wohnen aufgewendet werden. Unterstellt man vereinfachend je Person eine fixe Budgetgrenze für dieses Gut, wäre bei steigenden Preisen die Fläche pro Person rückläufig, was eine höhere Nachfrage nach kleineren Wohnungen begünstigen würde.

### Erlösanalyse aggregierter Daten

Die Veränderung der Wohnungsgröße hat insbesondere dann einen Einfluss auf die erzielten Mieterlöse, wenn unterschiedliche Wohnungsgrößen zu unterschiedlichen Mieterträgen pro Quadratmeter führen, also kein proportionaler Zusammenhang zwischen absoluter Miete und Größe besteht. Legt man zunächst die vereinfachende Annahme zu Grunde, dass die Gesamtwohnfläche eines Gebäudes bei einer Aufteilung in kleinere Einheiten gleichbleibt, bedeutet dies, dass es bei nicht konstanten Quadratmetermieten eine erlösmaximierende Wohnungsgrößenaufteilung gibt. Die Vereinfachung der Flächenaufteilung wird in Kap. 4 berücksichtigt und aufgehoben. Als Datengrundlage werden zur Überprüfung des Zusammenhangs von Wohnungsgröße und Quadratmetermiete auf Grund der Verfügbarkeit Angebots- sowie Mietspiegeldaten aus der Wissenschaftsstadt Darmstadt, ebenfalls innerhalb des Untersuchungsraums Rhein-Main, herangezogen.

**Figure 4:**
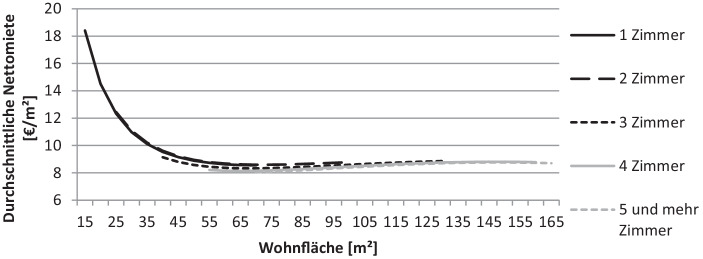

Die Visualisierung der Mietspiegeldaten in Abb. 4 zeigt den in der Theorie bekannten, abnehmenden Verlauf der Quadratmetermiete in Abhängigkeit von der Wohnungsgröße und widerspricht somit einer konstanten Entwicklung. Weitere Wohnungscharakteristika wie Lage und Größe der Wohnung werden in diesem Fall über Zu- und Abschläge integriert.

Diese Erkenntnisse basieren auf den vergangenheitsbezogenen Bestandsmieten des Mietspiegels sowie üblicherweise auf der Verwendung von Regressionsmodellen, welche die funktionale Form vorgeben. Eine Analyse der Angebotsdaten aus dem Untersuchungszeitraum vom 28.01.2019 bis zum 19.08.2019 sollen die zukunftsbezogeneren Angebotsdaten auf ähnliche Zusammenhänge prüfen.

### Erlösanalyse und Prüfung der Erkenntnisse an aktuellen Marktdaten

Zur Validierung der im Mietspiegel identifizierten Zusammenhänge wurden für die Wissenschaftsstadt Darmstadt vorliegende Angebotsdaten bereinigt und aufbereitet. Die Angebote wurden über die Postleitzahl als eindeutiges Lagekriterium ausgewählt und Angebote für möblierte Wohnungen, für WG-Zimmer, zur Zwischenmiete, in Wohnheimen und für Serviced Apartments aus dem Datensatz entfernt. Des Weiteren wurden Duplikate- und Ausreißer-Prüfungen durchgeführt. Nach der Bereinigung umfasst der Datensatz knapp 1000 Angebote.

Für die Datenanalyse werden die Inserate nach deren Wohnfläche in Cluster eingeteilt. Die Durchschnittskaltmieten je m^2^ Wohnfläche der einzelnen Cluster werden in Diagrammen durch miteinander verbundene Punkte visualisiert, um die Lesbarkeit des Trends zu vereinfachen. Darüber hinaus werden die Quadratmetermieten eines jeden Clusters durch Box-Plots visualisiert. Diese geben für jedes Cluster das 1., 2, (Median) und 3. Quartil sowie das Minimum und das Maximum an. So lassen sich aus den Diagrammen nicht nur Durchschnittswerte, sondern auch Informationen über die Streuung der Werte der einzelnen Cluster ablesen. Außerdem ist für jedes Cluster die Anzahl der enthaltenen Inserate angegeben, um die Belastbarkeit der Durchschnitts- und Quartilswerte der Cluster beurteilen zu können.

**Figure 5:**
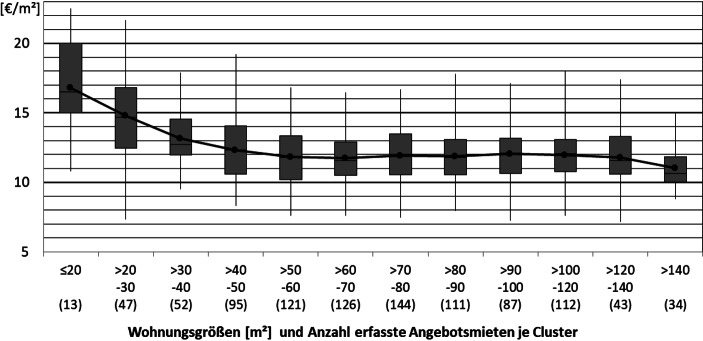

Aus Abb. 5 geht hervor, dass bei den erfassten Angebotsmieten bei den Wohnungsgrößenclustern bis 60 m^2^ ein deutlich negativer Zusammenhang zwischen der Wohnfläche der Wohnungen und der durchschnittlichen Kaltmiete je m^2^ Wohnfläche festgestellt werden kann. Dieser Zusammenhang fällt mit steigenden Wohnungsgrößen weniger stark aus. Die durchschnittlichen Angebotsmieten der Wohnungsgrößencluster mit über 70 bis 120 m^2^ bewegen sich, mit leichten Schwankungen, um ca. 12 €/m^2^ und somit, entgegen dem festgestellten Trend, leicht über dem Durchschnitt der Angebotsmieten von Wohnungen mit 60–70 m^2^. Die durchschnittlichen Quadratmetermieten sinken bei den Wohnungsgrößenclustern ab 90 m^2^ bei steigenden Wohnungsgrößen wieder, allerdings unterschreiten erst die Quadratmetermieten der Wohnungen mit mehr als 140 m^2^ Wohnfläche das zwischenzeitlich erreichte lokale Minimum des Größenclusters von 60–70 m^2^. Dass die Quadratmetermieten von Wohnungen mit ca. 70 bis 120 m^2^ mit steigenden Wohnungsgrößen nicht weiter sinken, sondern im Vergleich zum 60–70 m^2^ Cluster sogar leicht ansteigen, könnte auf einen höheren Anteil besser ausgestatteter Wohnungen oder auf die spezielle Nachfragesituation in der entsprechenden Lage zurückzuführen sein, da insbesondere in sehr guten Lagen teils die Umkehrung des klassischen Zusammenhangs beobachtet werden kann (Kleiber et al. 2017). Der beschriebene Verlauf spiegelt sich qualitativ in ähnlicher Form auch in den Verläufen der Quartilswerte (1., 2. und 3. Quartil) wieder. Bei der Betrachtung der Quartilswerte fällt darüber hinaus eine große Spanne zwischen dem 1. Quartil und dem 3. Quartil bei den beiden kleinsten Größenclustern auf, was auf eine relativ breite Streuung der Quadratmetermieten in diesen Clustern schließen lässt.

Die Analyse anhand des Darmstädter Mietspiegels plausibilisiert den Verlauf der Miethöhen in Abb. 5 qualitativ, allerdings auf einem deutlich (ca. 3 €/m^2^) niedrigerem Mietniveau, das durch die Konstruktion des Mietspiegels bedingt sein könnte. Der Mietspiegel basiert nicht auf Angebotsmieten und umfasst Mietangaben, die bis zu 4 Jahre zurückliegen. Darüber hinaus erfasst der Mietspiegel neben Neuvertragsmieten auch Mieten, die sich in den vergangenen Jahren durch Mieterhöhungen (hierzu zu zählen auch „automatische“ Mieterhöhungen in Staffel- und Indexmietverträgen) verändert haben (Bundesamt für Bauwesen und Raumordnung 2014). Es kann davon ausgegangen werden, dass diese Mieten, auch nach den automatischen Mieterhöhungen, unter der Berücksichtigung der Mietpreisentwicklungen der Jahre 2015 bis 2019, im Durchschnitt noch unterhalb möglicher Neuvertragsmieten für die gleichen Wohnungen liegen. Obwohl Gefälligkeitsmietverträge im Mietspiegel nicht berücksichtigt werden sollen, ist davon auszugehen, dass der Mietenmaximierung bei inserierten Mietangebote, z. B. in Onlineportalen, eine höhere Bedeutung zukommt, als bei privat vermittelten Wohnungen. Letztere sind im Mietspiegel enthalten, in einer auf Daten aus Immobilienportalen basierenden Auswertung allerdings nicht.

Um zu überprüfen, ob dem mittels deskriptiver Statistik festgestellten negativen Zusammenhang zwischen Wohnungsgröße und Miethöhe auch eine direkte Kausalität zu Grunde liegt, wurden im Folgenden weitere Analysen durchgeführt. Bei diesen wurden Ausschnitte des Datensatzes betrachtet und/oder kalkulatorische Abzüge für in den Mieten inkludierten Stellplätzen oder Einbauküchen angesetzt. Durch die zusätzlichen Fokussierungen in den beschriebenen Analysen verkleinert sich jeweils die Datenbasis. Es kann aber kein Hinweis darauf gefunden werden, dass der festgestellte Zusammenhang zwischen Wohnungsgröße und Miethöhe zurückzuführen wäre auf:

- eine Korrelation zwischen Lagequalität und Wohnungsgröße
- eine Korrelation zwischen Baujahr und Wohnungsgröße
- auf die Mieter umgelegte Kosten für Stellplätze oder Einbauküchen

**Figure 6:**
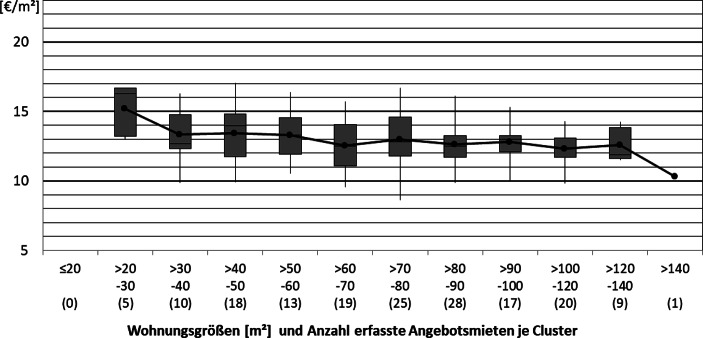

Gegenteilig bestärkt die Veränderung der Kurven bei einem Ausschluss älterer Wohnungen sowie Wohnungen mit luxuriöser Ausstattung und Penthouses die Vermutung eines Luxus-Effektes in den mittleren Größenclustern. Der Zusammenhang zwischen Wohnungsgröße und Miethöhe unter Berücksichtigung obiger Einschränkungen ist in Abb. 6 dargestellt. Dabei wurden die Stadtteile Eberstadt und Arheilgen aufgrund einer etwas periphereren Lage ausgeschlossen. Die Lincoln-Siedlung (eine Quartiers-Entwicklung im Süden Darmstadts) wurde, aufgrund der Vielzahl der aus ebendieser stammenden Angebote, welche alle von demselben kommunalen Entwickler stammen, ausgeschlossen. Zudem wurden nur Neubauten und neubauähnliche Wohnungen betrachtet, da deren Angebotsmieten für die Markteinschätzung bei Neubau-Projektentwicklungen am wesentlichsten sind.

Die Darstellung in Abb. 6 basiert aufgrund der Fokussierung auf Teilmärkte auf einer geringeren Anzahl an Datenpunkten. Dennoch zeigt sich, dass die Fokussierung bezüglich Lage, Ausstattung und Baujahr gerade nicht dazu führt, dass der Zusammenhang zwischen Wohnungsgröße und Miethöhe nicht mehr zu erkennen wäre.

**Figure 7:**
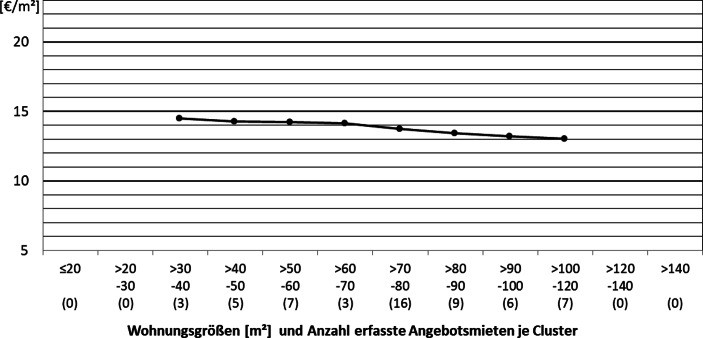

In Abb. 7 ist ein deutlicher Zusammenhang zwischen Wohnungsgröße und Miethöhe über alle Wohnungsgrößencluster, in denen sich Neubauwohnungen aus der Lincoln-Siedlung finden, hinweg erkennbar. Einerseits ist dabei zwar zu beachten, dass diese Angebotsmieten vermutlich alle aus der gleichen Kalkulation des einheitlichen Investors stammen, weshalb die Box-Plots nicht mehr als solche zu erkennen sind. Andererseits sind die Wohnungen bezüglich ihrer Lage, ihres Baujahrs, und ihrer Ausstattung aber sehr gut miteinander vergleichbar. Darüber hinaus sind die Wohnungen weder mit Einbauküchen ausgestattet, noch sind in den Kaltmieten Stellplätze inkludiert. Somit zeigt sich, dass der Vermieter hier davon ausgeht, für kleinere Wohnungen höhere Quadratmetermieten erzielen zu können.

Insgesamt kann festgestellt werden, dass sowohl die Analyse des Mietspiegels wie auch die Auswertung der Angebotsdaten starke Indizien dafür liefern, dass mit der Entwicklung kleinerer Wohnungen höhere Mieten pro Quadratmeter erzielt werden können, da für beide Datengrundlagen aus der Wissenschaftsstadt Darmstadt eine mit der Wohnungsgröße fallende Quadratmetermiete beobachtet werden kann. Insbesondere für Wohnungen kleiner 60 m^2^ wird dieser Effekt gut in den Abb. 4 und 5 deutlich. Abschließend bleibt festzuhalten, dass es sich bei dieser Betrachtung nur um eine Betrachtung der aktuellen Situation handeln kann. Am Modell von DiPasquale und Wheaton (1992) wird beispielsweise deutlich, dass durch die komplexen Zusammenhänge auf verschiedensten Märkten Veränderungen von Angebotsvariablen, wie dem Gesamtbestand und der damit verknüpften Neubauaktivität, aber auch von Nachfragevariablen, zu grundsätzlich anderen Mieten und Mietzusammenhängen führen können.

## Negative Effekte durch die Entwicklung kleinerer Wohnungen auf die Kosten und Erlöse einer Projektentwicklung

Neben dem positiven Aspekt höherer zu erwartender Mieterträge je Quadratmeter Wohnfläche, gehen mit der Entwicklung kleinerer Wohnungen aber auch Nachteile für Entwickler einher. Zur Bestimmung eben dieser Nachteile ist keine standardisierte Methodik bekannt, auf der aufgebaut werden kann. Daher wurde zur Strukturierung eine Analyse der wesentlichen Erlös- und Kostenpositionen mit Hilfe der einfachen Developer-Rechnung durchgeführt, da diese alle relevanten Einflüsse einer Projektentwicklung abbilden sollte. Hierzu wurden zunächst wesentliche Abhängigkeiten zwischen der Wohnungsgröße und den Unterpositionen der Kosten ermittelt, indem jede Position einer individuellen Analyse anhand gängiger Normen und Tabellenwerke, wie der DIN 276, den NHK 2010 oder den Kostenkennwerten des Baukosteninformationszentrums Deutscher Architektenkammern, in Bezug auf eine Größenabhängigkeit unterzogen wurde. Diese Werke ermöglichen entweder eine feinere Untergliederung der einzelnen Positionen in ihre Teilpositionen oder liefern Umrechnungskoeffizienten für die Größe, wodurch Abhängigkeiten deutlicher werden. Diese Analyse der einzelnen Positionen wurde durch Interviews ergänzt, um die Ergebnisse zu plausibilisieren und sicher zu stellen, dass keine wesentlichen Aspekte aus der Praxis unberücksichtigt bleiben. Da es für viele Positionen, wie beispielsweise die Grundstückskosten und Grunderwerbsnebenkosten offensichtlich oder zumindest naheliegend ist, dass es keine oder nur eine geringe Abhängigkeit von der Wohnungsgröße gibt, wurden lediglich die in der Recherche und durch die Experten bestätigten, wesentlichsten Positionen ausführlich diskutiert. Diese werden anschließend an die Beschreibung der einfachen Developer-Rechnung in Abschn. 4.1 in den Abschn. 4.2–4.4 erläutert. Eine zusammenfassende Darstellung der Ergebnisse aller Positionen der einfachen Developer-Rechnung findet sich in Tab. 2 am Ende des Kapitels.

### Struktur der Kostenanalyse und Auswahl wesentlicher Parameter

Im Folgendem sind zunächst die Kosten und Erlöse eines Projektentwicklers gemäß der einfachen Developer-Rechnung in Anlehnung an Schulte et al. (2002) dargestellt.

**Mieteinnahmen pro Jahr**

**× Verkaufsfaktor**

**− Kosten:**

1. Grundstücks- und Grunderwerbsnebenkosten
2. Bau- und Baunebenkosten
3. Kosten Projektmanagement (ohne Leistungen Projektenwickler)
4. Unvorhergesehenes
5. Marketingkosten
6. Vermietungskosten bzw. Maklerprovision
7. Zwischenfinanzierung des Grundstücks
8. Zwischenfinanzierung der Positionen 2, 4 und 5
9. Zwischenfinanzierung von Mietausfällen aufgrund anfänglichen Leerstands

**= Trading Profit**

Bei der einfachen Developer-Rechnung ergibt sich der Trading Profit des Projektentwicklers aus der Differenz zwischen dem Verkaufspreis des Objektes und den dem Projektentwickler entstehenden diversen Investitionskosten. Der Verkaufspreis ergibt sich aus den jährlichen Mieteinnahmen und dem Verkaufsfaktor. Der Verkaufsfaktor gibt dabei an, das wievielfache der jährlichen Mieteinnahmen ein Endinvestor voraussichtlich für das geplante Objekt zu zahlen bereit ist (Schulte et al. 2002). Der diesem Faktor zugrundeliegende Zusammenhang zwischen Immobilieninvestmentmarkt und Mietflächenmarkt ist insbesondere von der erwarteten Sicherheit der Mieterträge, der Attraktivität alternativer Anlagemöglichkeiten und der Erwartung an die zukünftige Entwicklung der Mieterträge abhängig (DiPasquale und Wheaton 1992).

Der voraussichtlich erzielbare Trading Profit ist für den Projektentwickler ein wesentliches Entscheidungskriterium für oder gegen die Durchführung eines Projektes. Neben individuellen Parametern, wie beispielsweise der aktuellen Auslastung oder der Attraktivität alternativer Projektentwicklungsmöglichkeiten, können insbesondere die Eigenkapitalverzinsung, das kalkulierte Risiko sowie die notwendigen eigenen Kapitalmittel für die Bewertung des voraussichtlichen Trading Profits von Relevanz sein (Schulte et al. 2002).

Mittels der in der Einleitung des vierten Kapitels beschriebenen Methodik konnten die Mieteinnahmen pro Jahr, über die sinkende Flächeneffizienz, und die Kostenposition Bau- und Baunebenkosten als wesentlichste Positionen identifiziert werden, die im Weiteren beschrieben werden. Die Bau- und Baunebenkosten werden nochmals unterteilt in die direkt mit der Planung kleinerer Wohnungen einhergehenden Bau- und Baunebenkosten und die oftmals damit in Verbindung stehenden Kostensteigerungen auf Grund einer höheren Stellplatzanzahl.

### Sinkende Flächeneffizienz durch die Planung kleinerer Wohnungen

Die folgenden Gründe begünstigen, bei einer höheren Anzahl an Wohnungen auf gleicher Grundfläche, eine sich verringernde Flächeneffizienz (Wohnfläche/Bruttogrundfläche):

- ein steigender Anteil an Wohnungstrennwänden und gegebenenfalls Wohnungsinnenwänden
- ein größerer Flächenanteil für Schächte der Haustechnik
- eine höhere Anzahl an Treppenhauskernen oder mehr Flächen für Hausflure

Ob die genannten Aspekte relevant sind und in welchem Ausmaß ist stark einzelfallabhängig. Mit einem Treppenhauskern lassen sich ohne zusätzliche Flure in der Regel drei bis maximal vier Wohnungen erschließen. Vergleicht man eine Planung mit kleineren Wohnungen und Dreispännern mit einer Alternative mit größeren Wohnungen und Zweispännern würden in diesem Fall kleinere Wohnungen noch nicht zu einem Flächeneffizienzverlust durch zusätzlich notwendige Treppenhauskerne führen. Daher ist eine Quantifizierung, auch in Bandbreiten, aus Expertensicht nicht sinnvoll und es kann nur der grundsätzlich negative Zusammenhang festgestellt werden.

### Höhere Baukosten durch die Planung kleinerer Wohnungen

Die Baukosten betreffen unterschiedlichste Bereiche. Zur Untersuchung des Einflusses der Entwicklung kleinerer Wohnungen auf die Baukosten eines Projektes bietet sich daher die Aufschlüsselung der Projektkosten nach der DIN 276, die regelmäßig zur Ermittlung der Projektkosten herangezogen wird an, um eine strukturierte Analyse zu ermöglichen. In nachfolgender Tab. 1 sind mögliche Auswirkungen aufgeführt, die aus Sicht der Autoren die Baukosten erhöhen könnten. Die Individualität von Wohnbauprojekten und der einzelnen Positionen innerhalb dieser Projekte lässt eine exakte Quantifizierung allerdings nicht zu.

**Table 1:** 

Kostengruppen	Mögliche Steigerung der Baukosten durch
*300*	
330, 340	Eine höhere Anzahl an Wohnungs‑, Raum,- und Balkontüren sowie Fenstern (inkl. Lichtschutz)
	Eine größere Gesamtfläche mit teurerer Wandbekleidung (Fliesen) durch eine höhere Anzahl an Bädern und Küchen (falls letztere eine teurere Wandbekleidung aufweisen)
	Eine höhere Anzahl an Wohnungstrenn- und Wohnungsinnenwänden
350	Eine höhere Anzahl an Treppenhäusern, um eine höhere Anzahl an Wohnungen zu erschließen
	Eine höhere Anzahl an Balkonen (auch bei gleicher Gesamtbalkonfläche)
	Eine größere Gesamtfläche mit teureren Deckenbelägen (Fliesen) durch eine höhere Anzahl an Bädern und Küchen
380	Eine höhere Anzahl an Einbauküchen
390	Eine höhere Anzahl an TGA-Schächten aufgrund einer höheren Anzahl an Bädern und Küchen
	Höhere Kosten für Schließanlagen aufgrund einer höheren Anzahl an Wohnungen
390, 490, 590	Eine gegebenenfalls leicht verlängerte Bauzeit, die die Kosten für das Vorhalten der Gerüste und der Baustelleneinrichtung sowie die Baustellengemeinkosten erhöhen könnte
*400*	
410	Höhere Kosten für Wasser- und Abwasseranlagen aufgrund einer höheren Anzahl an Küchen und Bädern
420	Höhere erforderliche Kapazitäten der Wassererwärmungsanlagen aufgrund einer höheren Anzahl an Wohnungen und gegebenenfalls Bewohnern
	Eine höhere Anzahl an Verteilern, Rohrleitungen und Pumpen
	Eine höhere Anzahl an Raumheizflächen aufgrund einer höheren Anzahl an Räumen
430	Eine höhere Anzahl an Lüftungs- oder Klimaanlagen und eine größere Menge an Zu- und Abluftleitungen
440	Eine höhere Anzahl an Verteilern, Unterverteilern, Kabeln und Leitungen für elektrische Anlagen
450	Höhere Kosten für Telekommunikationsanlagen, Klingel‑, Türsprech- und Türöffneranlagen, sowie Fernseh- und Antennenanlagen
460	Eine höhere Anzahl an Aufzügen
*700*	
710	Höhere Kosten für Bauherrenaufgaben aufgrund komplexerer oder zusätzlicher Bauabläufe
720, 730	Eine umfangreichere oder komplexere Objektplanung
740	Umfangreicherer oder komplexerer Fachplanungen (TGA, Brandschutz)
760	Beispielsweise höhere Bewirtschaftungskosten für die Baustellenbüros für Bauherren und Planer aufgrund einer gegebenenfalls leicht verlängerten Bauzeit
*Kostengruppenübergreifend*	Eine höhere Anzahl an Außenstellplätzen und/oder Tiefgaragenstellplätzen, die allerdings übergreifend erst in Abschn. 4.4 bewertet wird

Auf Grundlage der in Tab. 1 dargestellten Aufstellung wurden zunächst die folgenden Punkte als die wesentlichen Kostentreiber bei einer Verringerung der Wohnungsgrößen identifiziert:

- höherer Flächenanteil mit teureren Wandbekleidungen bzw. Bodenbelägen (Fliesen) und eine höhere Anzahl an Wasseranschlüssen aufgrund einer höheren Anzahl an Bädern und Küchen
- eine höhere Anzahl an Türen und Fenstern
- eine höhere Anzahl an Innenwänden
- eine komplexere und umfangreichere TGA
- eine höhere Anzahl an Treppenhäusern

Um diese Hypothese zu plausibilisieren, wurden Einschätzungen von Baukostenexperten herangezogen. Dabei wurden von allen Interviewpartnern höhere TGA-Kosten als am wesentlichsten angegeben. Als Gründe hierfür wurden beispielhaft eine höhere Anzahl an Zählern, Leuchtenauslässen, Sicherungen, Schaltern, Steckdosen, Kabeln, Wasserzuläufen und Wasserabläufen aufgezählt. Die vermuteten Einflüsse einer höheren Anzahl an Bädern, Küchen und Innenwänden wurden von den Interviewpartnern im Wesentlichen bestätigt. Dabei wurde darauf hingewiesen, dass Bäder aufgrund von Wasseranschlüssen, Fliesen und Badgegenständen in der Regel entscheidender seien als Küchen. Der Einfluss einer höheren Anzahl an Türen wurde hingegen als weniger relevant eingeschätzt. Als Empfehlung zur Minimierung von Baukostensteigerungen bei der Entwicklung kleinerer Wohnungen wurde dabei stets angemerkt, dass die Anzahl an Treppenhäusern möglichst gering gehalten werden sollte.

### Höhere Stellplatzanzahl durch die Planung kleinerer Wohnungen

Die Planung kleinerer Wohnungen und somit einer höheren Anzahl an Wohnungen auf gleicher Fläche, führt einerseits zu einer höheren Anzahl an Stellplätzen und hiermit einhergehend zu höheren Herstellungskosten, andererseits erzielen zusätzliche Stellplätze in der Regel auch zusätzliche Erlöse. Zudem sind die Herstellungskosten und die Erlöse stark einzelprojektabhängig und zusätzliche oberirdische Stellplätze benötigen freie Grundstücksfläche, die in aller Regel knapp ist. Aus diesen Gründen wurden die Errichtungskosten für Stellplätze im vorherigen Abschnitt ausgeklammert. Stattdesessen wird in diesem Abschnitt der Einfluss auf die Kosten und Erlöse eines Projektes insgesamt qualitativ diskutiert.

Bei einer Erhöhung der Wohnungsanzahl eines Projektes steigt der Bedarf an Stellplätzen aufgrund einer höheren Anzahl an Haushalten und gegebenenfalls höheren Anzahl an Bewohnern. In Abhängigkeit von der Verfügbarkeit von Parkplätzen in der Umgebung des Projektes könnte eine höhere Anzahl an Wohnungen zu einer intrinsischen Motivation des Projektentwicklers führen, eine höhere Anzahl an Stellplätzen vorzusehen, da sich zusätzliche Erlöse erzielen lassen könnten. Zum anderen kann eine zu geringe Anzahl an Stellplätzen die Vermarktbarkeit der Wohnungen verschlechtern. Darüber hinaus können Gemeinden festlegen, dass bei der Errichtung von Wohngebäuden Stellplätze herzustellen sind. Für kleinere Wohnungen werden zwar teils weniger Stellplätze je Wohnung gefordert als bei größeren Wohnungen. Regelmäßig führt die Planung kleinerer Wohnungen, was gleichbedeutend mit einer höheren Wohnungsanzahl ist, aber zu einer höheren Anzahl herzustellender Stellplätze. Aufgrund der beschriebenen Komplexität und Einzelfallabhängigkeit des Einflusses der Herstellung von Stellplätzen auf die Kosten und Erlöse eines Projektes werden ebenfalls Expertenmeinungen miteinbezogen. Gemäß dieser ist die Errichtung von Stellplätzen in der Praxis sehr häufig ein Zuschussgeschäft bzw. im günstigsten Fall kostenneutral. Insbesondere die Herstellung von Tiefgaragenstellplätzen ist nicht durch deren Mieterlöse zu finanzieren. Darüber hinaus wird bestätigt, dass die Herstellung von Außenstellplätzen wesentlich günstiger sei, allerdings auch geringere Mieterlöse generiere, die Quartiersqualität insgesamt verringern könne und unter Umständen baurechtlich oder aufgrund der endlichen Grundstücksfläche nicht möglich sei. Aus den dargestellten Gründen geben alle Experten an, dass in der Praxis von einem negativen Einfluss der Entwicklung kleinerer Wohnungsgrößen durch die Herstellung einer höheren Anzahl an Stellplätzen auf den Trading Profit auszugehen sei.

Tab. 2 stellt zusammenfassend und an der Struktur der Developer-Rechnung orientiert die ermittelten Einflüsse der Entwicklung kleinerer Wohnungen auf alle Positionen dar. Die dargestellten Effekte wirken sich in Summe auf die Höhe des Trading Profits eines Projektes aus.

**Table 2:** 

Betrachtete Aspekte	Erlöse	Kosten	Kurzbegründung
*Mieten je m*^*2*^* Wohnfläche*	**↗**	–	Es wird ein grundsätzlich negativer Zusammenhang zwischen Wohnungsgröße und Quadratmetermiete festgestellt
*Flächeneffizienz*	**↘**	–	Es wurden begründete Vermutungen für einen positiven Zusammenhang zwischen Wohnungsgrößen und Flächeneffizienz aufgestellt und durch Experten bestätigt
*Verkaufsfaktor*	–	–	Die interviewten Experten sehen in der Praxis keinen Einfluss der Wohnungsgrößen auf den Verkaufsfaktor
*Grundstücks- und Grunderwerbsnebenkosten*	–	–	Es wurde die Vermutung formuliert, dass eine höhere Anzahl zulässiger Wohnungen mit einem höheren Kaufpreis einhergehen könnte, was von den interviewten Experten aber als praxisfern eingestuft wird. Außerdem könnten die Erschließungskosten leicht ansteigen. Insgesamt wird der Einfluss auf die Investitionskosten aber als nicht vorhanden bis als in der Regel vernachlässigbar gering eingeschätzt
*Bau- und Baunebenkosten (inkl. Projektmanagement und Pauschale für Unvorhergesehenes, ohne Stellplätze)*	–	↗	Die Baukosten erhöhen sich, was insbesondere auf eine höhere Anzahl an Bädern, Küchen, Wohnungstrennwänden und gegebenenfalls Treppenhäusern zurückzuführen ist. Insofern eine Pauschale für Unvorhergesehenes berücksichtigt wird, steigt diese vermutlich linear mit den Baukosten
*Stell- und Abstellplätze*	↗	**↗**	Gemäß den Experteninterviews übersteigen die Kosten der Errichtung von Stellplätzen in der Praxis meist deren direkte Erlöse. Da eine höhere Anzahl an Wohnungen die herzustellende Anzahl an Stellplätzen üblicherweise erhöht, ist von einem negativen Einfluss der Entwicklung kleinerer Wohnungsgrößen durch die Herstellung einer höheren Anzahl an Stellplätzen auf den Trading Profit auszugehen
*Marketing- und Vermietungskosten*	–	–	Insbesondere die Maklercourtage könnte sich bei einer höheren Anzahl an Wohnungen erhöhen. Für einen Einfluss auf den Verkaufsfaktor müssten die Kosten dabei überproportional zu den Mieterträgen ansteigen, was in der Praxis nicht üblich ist
*Zwischenfinanzierung von Grundstücks‑, Bau- und Marketingkosten*	–	**↗**	Die Finanzierungskosten erhöhen sich in der Regel proportional zu den zu finanzierenden Kosten. Ein Einfluss auf die Finanzierungskosten durch eine Verlängerung des Finanzierungszeitraums aufgrund der Entwicklung kleinerer Wohnungen wurde in den Experteninterviews hingegen als sehr gering eingeschätzt
*Zwischenfinanzierung von Mietausfällen aufgrund von anfänglichem Leerstand*	–	–	Die Kosten erhöhen sich typischerweise proportional zu den steigenden Mieterträgen, da bei einer höheren Anzahl an Wohnungen von einem ähnlichen durchschnittlichen Leerstand ausgegangen werden kann

## Zusammenfassung der Ergebnisse

### Wohnungsgrößenentwicklung

Es gibt bei der Entwicklung neuer Wohnungen einen Trend hin zu kleineren Einheiten, der an der rückläufigen Wohnfläche je Baugenehmigung in den letzten Jahren deutlich wird. Dabei ist zu beachten, dass auch Effekte wie die Urbanisierung und der Trend hin zu kleineren Haushaltsgrößen einen Einfluss haben und eine durchschnittliche Neubauwohnung noch immer über der Größe einer durchschnittlichen Bestandswohnung liegt.

### Mietpreishöhe in Abhängigkeit der Wohnungsgröße

Die Analyse der bestandsorientierten Mietspiegeldaten sowie der stärker zukunftsorientierten Angebotsdaten zeigen gleichermaßen, dass mit sinkender Wohnungsgröße für die betrachteten Fallbeispiele eine steigende Quadratmetermiete erzielt werden kann. Dieser Effekt tritt vor allem bei Wohnungen mit einer Größe kleiner 60 m^2^ auf.

### Negative Effekte auf Projektentwicklungen

Basierend auf Experteneinschätzungen aus der Baupraxis und Projektentwicklung zu den Bestandteilen der einfachen Developer-Rechnung, haben vor allem die abnehmende Flächeneffizienz sowie die steigenden Baukosten einen negativen Einfluss auf das Ergebnis eines Projektentwicklers. Bei den steigenden Baukosten gilt insbesondere den Stellplätzen und den Kosten auf Grund zusätzlicher TGA ein besonderes Augenmerk.

### Implikationen

Projektentwickler erwarten, gemäß den Aussagen der Praxisexperten, bei einem Projekt mit kleineren Wohnungsgrößen den gleichen prozentualen Aufschlag auf die Investitionskosten als Trading Profit. Dementsprechend müssten sich die Mieterträge eines Projektes durch die Entwicklung kleinerer Wohneinheiten, trotz der in der Regel leicht geringeren Flächeneffizienz und einer höheren Anzahl herzustellender Stellplätze, prozentual mindestens so stark erhöhen, wie es der Kostensteigerungen entspricht, dass ein Projekt durch die Entwicklung kleinerer Wohnungen für einen Projektentwickler attraktiver wird. Außerdem muss die Vermarktbarkeit bzw. Absorptionsfähigkeit für die zu entwickelnde Anzahl kleinerer Wohnungen gegeben sein. Diese ist in der Praxis nicht unbegrenzt. Die wohnungsgrößenabhängige Nachfrage ist außerdem standort- und einzelprojektabhängig. Die Analyse der Angebotsmietpreise in Abschn. 3.2 stellt somit beispielhaft die aktuelle Mietpreissituation für eine exemplarische Region dar und weist insbesondere bei kleinen Wohnungsgrößen bis ungefähr 60 m^2^ auf einen negativen Zusammenhang zwischen Wohnungsgröße und Miethöhe hin. Bei großen Entwicklungen werden Wohnungen unterschiedlicher Größe und Zimmeranzahl geplant, um das Risiko zu diversifizieren. Zusätzlich sprechen im zu Beginn beschriebenen Marktumfeld steigender Wohnraumpreise geringere absolute Mietpreise für die Entwicklung kleinere Wohnungen. Diese Einschätzung wird durch die Interviews bestätigt. Auf Grundlage der Betrachtungen dieser Arbeit und den durchgeführten Experteninterviews können zudem Handlungsempfehlungen für die Wohnungsgrößengestaltung gegeben werden, die an dieser Stelle abschließend gesammelt aufgezählt werden.

- Aus Kostengründen sollte bei der Entwicklung kleinerer Wohnungen versucht werden, die Anzahl der Treppenhauskerne möglichst gering zu halten, wobei bei der Abwägung gegen die Vorteile zusätzlicher Treppenhäuser, beispielsweise durch weniger oder keine Wohnflächenverluste durch Hausflure und höhere Mieterträge bei kleineren Wohnungen, im Einzelfall auch zusätzliche Treppenhäuser sinnvoll sein können.
- Da Stellplatzsatzungen häufig Wohnungsgrößengrenzen enthalten, sollten diese bereits in die frühen Planungsphasen einbezogen werden, um diese entsprechend berücksichtigen und gegebenenfalls die Stellplatzanzahl reduzieren zu können.
- Allgemein sollten neben dem positiven Effekt der steigenden Quadratmetermieten frühzeitig auch die negativen Auswirkungen kleinerer Wohnungsgrößen bei der Wohnungsgrößengestaltung berücksichtigt werden.

## Ausblick

Die zukünftige Entwicklung der Nachfrage nach kleineren Wohnungen ist von unterschiedlichen Faktoren abhängig. So könnten demographische Entwicklungen dazu führen, dass sich die durchschnittliche Wohnungsbelegung weiter reduziert (Statistisches Bundesamt 2019d). Gleichzeitig könnte eine Schrumpfung der Gesamtbevölkerung (Statistisches Bundesamt 2019b) die Nachfrage nach Wohnraum insgesamt reduzieren, was aufgrund von Urbanisierungsentwicklungen allerdings nicht zwangsläufig eine Reduzierung der Nachfrage in einzelnen angespannten regionalen Teilmärkten zur Folge hätte.

Aus den Experteninterviews geht zudem hervor, dass sich kleinere Wohnungen, und insbesondere deren Bäder, besser für eine Vorfertigung eignen als größere Wohnungen. Sollten sich die Baukosten, wie von vielen Akteuren erwartet oder zu mindestens erhofft, zukünftig durch die Digitalisierung und Industrialisierung von Bau- und Planungsprozessen verringern, könnte dies die Herstellkosten kleinerer Wohnungen relativ am stärksten reduzieren, was sich wiederum auf deren Mieten und der Nachfrage nach diesen auswirken könnte.

Des Weiteren könnten sich politische Maßnahmen zur Begrenzung des Klimawandels langfristig auch auf die Wohnungsgrößenentwicklung auswirken. So könnte die in Deutschland ab 2021 beginnende Bepreisung des Ausstoßes von CO_2_ in den Bereichen Verkehr und Wohnen (Presse- und Informationsamt der Bundesregierung 2019), je nach Höhe des Preises und Verwendung der Einnahmen, das verfügbare Einkommen von Mietern reduzieren und deren Wohnkosten erhöhen, was sich positiv auf die Nachfrage nach kleineren Wohnungen auswirken könnte. Eine hypothetisch gleiche Bepreisung wie die der Brenn- und Kraftstoffe, würde sich bei Mehrfamilienhausneubauten im Passivhaus-Standard im Vergleich zum EnEV-2016-Standard bei einer Differenz von etwa 40 kg CO_2_-Äquivalent je m^2^ Wohnfläche pro Jahr unter Berücksichtigung der Herstellungs- und Nutzungsphase (Mahler et al. 2019) und einem geplanten Einstiegspreis von 25 € je Tonne CO_2_ (Presse- und Informationsamt der Bundesregierung 2019), in einer Differenz von rund 1 € pro m^2^ Wohnfläche und Jahr auswirken.

Auch die COVID-19-Pandemie könnte die Nachfrage nach kleineren Wohnungen sowohl positiv als auch negativ beeinflussen, insofern diese sich nachhaltig auf Urbanisierungsprozesse, die Miet- und Kaufpreise für Wohnraum oder das für Wohnen verfügbare Einkommen auswirken sollte.
